# Effects of COVID19 Pandemic on Pediatric Kidney Transplant in the United States

**DOI:** 10.21203/rs.3.rs-72427/v1

**Published:** 2020-09-08

**Authors:** Olga Charnaya, Teresa Po-Yu Chiang, Richard Wang, Jennifer Motter, Brian Boyarsky, Elizabeth King, William Werbel, Christine M. Durand, Robin Avery, Dorry Segev, Allan Massie, Jacqueline Garonzik-Wang

**Affiliations:** Johns Hopkins University; Johns Hopkins University; Johns Hopkins University; Johns Hopkins University; Johns Hopkins University; Johns Hopkins University; Johns Hopkins University; Johns Hopkins University; Johns Hopkins University; Johns Hopkins University; Johns Hopkins University; Johns Hopkins University

**Keywords:** kidney transplantation, registry analysis, pediatrics, donation, infectious agents-viral

## Abstract

In March 2020, COVID-19 infections began to rise exponentially in the United States, placing substantial burden on the healthcare system. As a result, there was a rapid change in transplant practices and policies, with cessation of most procedures. Our goal was to understand changes to pediatric kidney transplantation (KT) at the national level during the COVID-19 epidemic. Using SRTR data, we examined changes in pediatric waitlist registration, waitlist removal or inactivation, and deceased donor and living donor (DDKT/LDKT) events during the start of the disease transmission in the United States compared to the same time the previous year. We saw an initial decrease in DDKT and LDKT by 47% and 82% compared to expected events and then a continual increase, with numbers reaching expected pre-pandemic levels by May 2020. In the early phase of the pandemic, waitlist inactivation and removals due to death or deteriorating condition rose above expected values by 152% and 189%, respectively. There was a statistically significant decrease in new waitlist additions (IRR _0.49_ 0.65 _0.85_) and LDKT (IRR _0.17_ 0.38 _0.84_) in states with high vs low COVID activity. Transplant recipients during the pandemic were more likely to have received a DDKT, but had similar cPRA, waitlist time and cause of ESRD as before the pandemic. The COVID-19 pandemic initially reduced access to kidney transplantation among pediatric patients in the United States, but has not had a sustained effect.

## Introduction

As the death toll from COVID-19 in the United States surpasses 180,000 fatalities, the pandemic continues to place considerable burden on the health care system. Many transplant centers have been challenged to determine how to proceed with organ transplantation during a time of limited data and resources. These decisions have had profound implications for many patients awaiting transplantation, including the pediatric population, where kidney transplantation (KT) remains the optimal treatment for end-stage renal disease (ESRD) [1].

Adult KT has been significantly impacted by COVID-19, with reduced new wait-list addition and transplantation at many transplant centers across the nation [2,3]. A national survey of adult transplant centers showed that 72% had suspended live donor kidney transplants (LDKT) and 84% had placed significant restrictions on deceased donor kidney transplants (DDKT) by April 2020 [4]. The stringency of these restrictions varied by center, with more restrictive measures in areas with a higher incidence of COVID-19. Several reasons have been cited for these changes. First, there is concern that organ transplant recipients may be at a greater risk for acquiring and experiencing worse clinical outcomes to COVID-19 infection [5–7]. Second, immunosuppressed KT recipients may have prolonged viral shedding and transmissibility, potentially posing a greater risk to public health [8,9]. Third, there is concern that hospitals will not have the resources to safely perform KT and provide the necessary post-operative care [10]. These changes are not without consequences; postponing KT can lead to a higher waitlist mortality, and diverting resources away from transplant recipients may worsen post-operative outcomes [11,12].

It is unclear if the pandemic has resulted in similar challenges and restrictions for the pediatric population. For the most part, children are asymptomatic or have milder infection and fewer complications from COVID-19 compared to adult patients [13,14]. From the limited data available, it appears that disease severity even among immunocompromised children remains less severe than that of the general adult population and remains similar to healthy children [15–17]. Furthermore, while pediatric patients in general have less dialysis-associated morbidity and mortality compared to adults, delaying transplantation and prolonging time on dialysis is associated with increased morbidity and repetitive risk of COVID exposure during in-center dialysis sessions [18,19]. The response of the pediatric transplant community is likely different than adult practices and therefore needs to be studied separately.

To investigate center-specific changes in pediatric transplant practices during the COVID-19 pandemic, we used national registry data to quantify changes to KT waitlist registration, waitlist deaths or removal, rates of DDKT and LDKT, between February and June 2020. We examined rates of these waitlist events among the overall patient population, and evaluated for differences in patient characteristics during the COVID-19 pandemic. This information will allow us to better understand of the pandemic’s impact on the pediatric kidney transplant population.

## Methods

### Data Source

This study used data from the Scientific Registry of Transplant Recipients (SRTR). The SRTR data system includes data on all donor, wait-listed candidates, and transplant recipients in the US, submitted by the members of the Organ Procurement and Transplantation Network (OPTN). The Health Resources and Services Administration (HRSA), U.S. Department of Health and Human Services provides oversight to the activities of the OPTN and SRTR contractors. This dataset has previously been described elsewhere [20].

### Study Population

For waitlist analysis, we included DDKT waitlist registrants aged 0–17 at time of listing (new listing; changed to inactive status; removed due to DDKT, LDKT, death or deteriorating condition, and other causes) were included. For analysis of transplant volume (DDKT, LDKT, DCD, national or regional import) recipients aged 0–17 at time of transplant were included.

### National cumulative incidence of COVID-19

National COVID-19 incidence data from January - June 2020 were extracted from USA FACTS (https://usafacts.org/issues/coronavirus/) [21]. COVID-19 incidence is reported for all patients and not restricted to children, as many states are not providing data of infections by age groups. Based upon cumulative incidence of COVID-19 positive cases per-million state population (PMP) between March 15 and June 30, states were stratified to be having low (<8000 PMP) or high (>8000 PMP) COVID-19 burden. This cutoff was selected based on an visible difference in distribution of cumulative incidence of COVID- 19 burden in the United States.

### Weekly counts of waitlist and transplant changes

For each week (Sunday-Saturday) starting February 2, 2020 until June 27, 2020, we plotted cumulative counts of new waitlist additions, newly inactive patients, waitlist removal due to death or deteriorating condition, and waitlist removal due to transplant or other causes, using a Lowess smoothing function. We made similar plots for weekly counts of DDKT, LDKT, DCD donor, and regional and national imports. Weekly instead of daily counts were used due to the low number of daily events in pediatric patients to enable statistical analysis. On each plot we also included the average counts during the same period in 2017–2019 as a visual reference of national pre-pandemic pediatric KT volume.

### Statistical analysis

We presented characteristics of pediatric kidney transplant recipients separately in three time periods: January 1 -March 15, 2020 (“Early”); March 16 - April 30, 2020 (“Middle”); and May 1 - June 30, 2020 (“Late”). Continuous variables were presented as median and interquartile range, and categorical variables were presented as counts and proportion. Comparison between groups were tested using Kuskal-Wallis or Mann Whitney-U, as appropriate, for continuous variables and Fisher’s exact test for categorical variables. We used 2015 as reference year to calculate KDPI [22]. We obtained pediatric kidney waitlist changes or transplant volume by center, month and year from January 1, 2016 to February 28, 2020, and constructed a mixed-effects Poisson regression with a center-level random intercept to obtain expected daily counts by center (monthly counts divided by 31), using methods previously described [23]. The expected counts of each time period were the sum of expected center-level counts during the corresponding length of time (March 1 to April 30: 47 days; May 1 - June 30: 61 days). We then compared the observed and expected counts of each time period using Chi-square testing. We used an a of 0.05 to define statistical significance. All analyses were performed using Stata 16.0/MP for Linux (College Station, Texas).

## Results

### Characteristics of pediatric transplant patients during COVID-19

Patient characteristics in three time periods (“Early” Jan 1 - Mar 15 2020, “Middle” Mar 16 - Apr 30 2020, and “Late” May 1 - Jun 30 2020) were examined (Table 1). Patients who received a kidney transplant during the first COVID-19 peak in the United States, Middle period, had similar waitlist time, cPRA and blood type compared to Early and Late periods (p > 0.1). Higher proportion of Black patients received a transplant in the Middle (30.6%) compared to Early (13.1 %) and Late (20.7%), (p=0.28). Living donor transplants made up a smaller proportion of total transplants during the Middle period 13.9%, compared to 29.5% and 36.4% in Early and Late, respectively (p=0.035). Median cold ischemia time (CIT) was longer in the Middle period 10.2 hours (IQR 6.5–17.4), compared to the Early 9.0 hours (IQR 4.0–13.2) and Late 7.6 hours (IQR 2.4–10.7) periods, (p=0.02).

### Weekly count of waitlist changes

**Figure 1:**
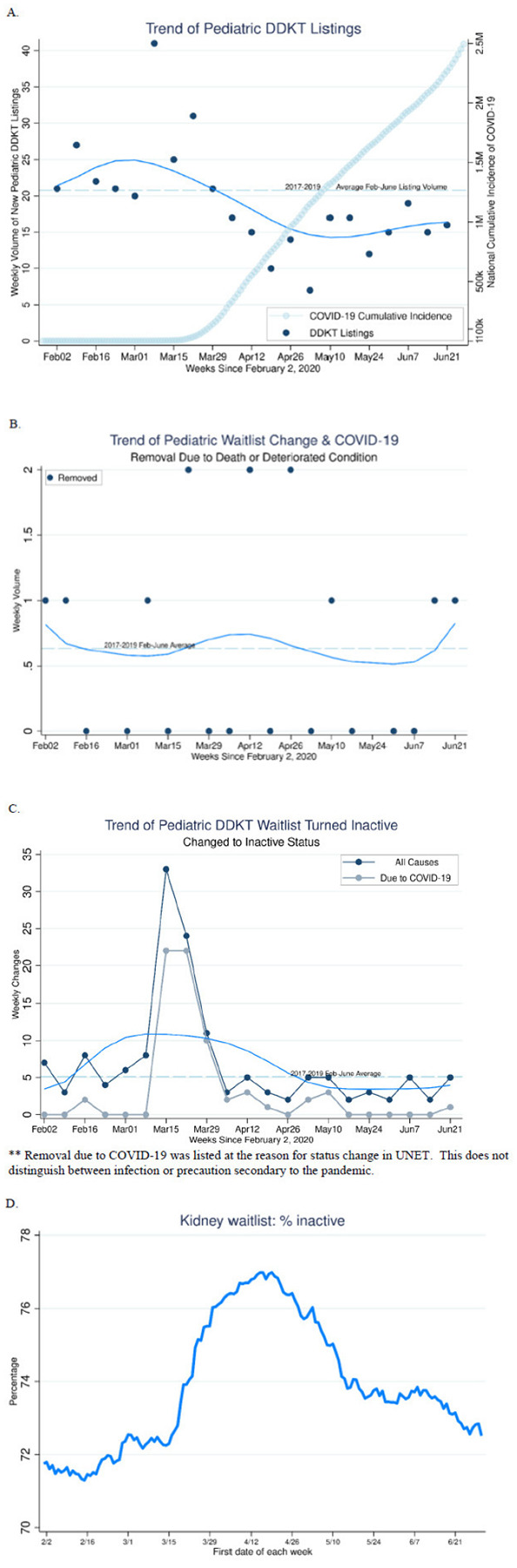
Pediatric patient deceased donor KT waitlist status change; (A) new waitlist additions, (B) removal due to death or deteriorating condition, (C) changed to inactive status, (D) percentage inactive. A.

National weekly pediatric KT waitlist additions ranged from 7 – 41 cases per week between February 2 and June 30, 2020. There was a trend of decreasing new pediatric DDKT registrations, following the national rise of COVID-19 cases mid-March. Since April, none of the weekly pediatric KTs exceeded 21, the 2017– 2019 average counts for the same period (Figure 1A). The numbers of registrants who changed to inactive status also increased in March, with 77.2% of registrants who changed to inactive status in the third and fourth week of March indicating COVID-19 as reason of inactivation (Figure 1C). COVID-19 was added as a refusal code or cause for change in status in UNET on March 25, 2020; however, this classification does not differentiate new COVID-19 infection in the patient vs precaution secondary to the pandemic.

Percentage of inactive waitlist registrants rose from 72% to 77% between March 1 and April 15, and remained elevated above previous baseline thereafter (Figure 1D). We observed an increasing trend in waitlist removal due to death or deteriorating condition since March, followed by a trend that returned to previous benchmarks by late-April (Figure 1B).

### Weekly count of transplant events

**Figure 2:**
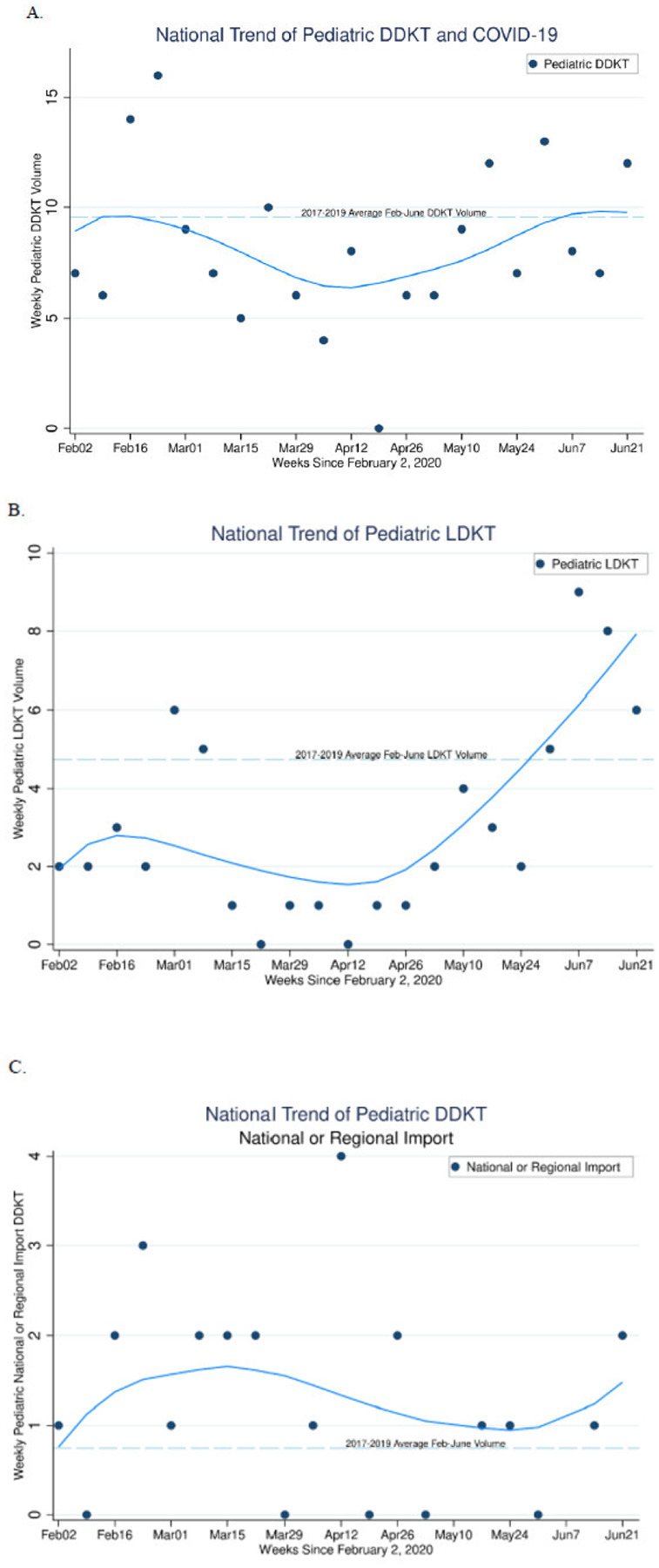
Pediatric transplant events cumulatively by week starting Feb 2, 2020; (A) DDKT, (B) LDKT, (C) Regional and National imports A.

The national weekly pediatric DDKT volume ranged from 0–16 cases per week between February 1 and June 30, 2020. On average, the weekly DDKT volume in 2017–2019 was 9.6 cases. Between mid-March and the end of June 2020, DDKT volume remained lower than 9.6 except for 4 weeks out of the 15 during observation. There was a trend of decreasing DDKT and LDKT volume seen since March, followed by increase from mid-April to end of June (Figure 2A, 2B). For LDKT, the weekly volumes were never above the 2017–2019 average between mid-March and May 31, but consistently surpassed this volume in June 2020 (Figure 2B).

### Regional and National Imports

Overall numbers of regional and national imports were extremely low (0–4 per week), with average <1 import per week in 2017–2019 (Figure 2C). During the early period of COVID-19 disease activity in the United States, imports were more common than in previous years. As the pandemic progressed, there was a decline in imports, however the average number of imports continue to remain higher than previous years.

### Comparing the observed and predicted waitlist changes

The overall observed national volume of waitlist registration was lower (−13.3%, p=0.021) and change to inactive waitlist status was higher (57.2%, p<0.001) compared to the expected volume during March 15 - June 30, 2020 (Table 2). When stratified into the earlier (March 15 - April 30, 2020) and the latter (May 1 - June 30, 2020) periods, 6 candidates were removed from the waitlist during the earlier period due to death or deteriorated condition, which was 189% more than the expected 2.1 cases (p=0.005). Similarly, 83 candidates had changed to inactive status during the earlier period, which was 152% more than the expected 32.9 cases (p<0.001). In both cases, the significance was not achieved in the latter period (11.3%, p=0.3 and −11.1%, p=0.5, respectively). Contrarily, the observed counts of new waitlist registration was 23.8% lower compared to the expected during the latter period (p=0.002), though not significantly different in the earlier period (0.4%, p=1.0).

### Comparing the observed and predicted transplant events

There were 157 pediatric KTs performed during March 15-June 30, 2020 (108 DDKT, 49 LDKT), which was 22.8% fewer than the expected 203.3 cases (p=0.001). The 108 DDKT performed during the same period was 29.2% fewer than the expected 103.1 cases (p=0.03), whereas the 49 LDKTs was not significantly different from the expected 64.2 cases (p=0.058). When stratified to the earlier and the latter COVID-19 eras, the observed DDKT, LDKT, and combined total transplant were all significantly less than expected in the earlier era (total: 36 vs. 88.5, −59.3%, p<0.001; LDKT: 5 vs. 27.9, −82.1%, p<0.001; DDKT: 31 vs. 59.1%, −47.6%, p<0.001) but not during the latter period.

### Regional differences in transplant practice and waitlist death by COVID-19 burden

Centers situated in states with high COVID-19 burden (NY, NJ, RI, MA, DC, CT,LA, DE, IL, MD, AZ, NE, IA, NS) between March 15 and June 30 had significantly fewer new waitlist registrations (incidence rate ratio (IRR): 0.49 0.65 0.85) and LDKT (IRR: 0.17 0.38 0.84) compared to centers in states with low burden (IRR: 0.82 0.94 1.08) (Table 3). There were no differences in the proportion of expected DDKT and waitlist death between centers in states with high and low COVID-19 burden.

## Discussion

In this national registry study of pediatric KT trends during the COVID-19 pandemic, we found an increase in patients being changed to inactive status by 152%, increase in mortality on the waitlist by 189%, decrease in DDKT by 48% and LDKT by 82% compared to expected in the early COVID-19 time period without a significant impact on new waitlist additions. The COVID-19 pandemic has substantially limited access to KT and increased waitlist mortality in pediatric patients.

Many transplant programs significantly altered their routine protocols and stopped performing kidney transplants thereby restricting access to KT during the COVID-19 pandemic [4]. A recent registry of adult SOT recipients infected with COIVD-19 did not show any significant difference in mortality or morbidity compared to non-SOT patients [24]. While extensive data in pediatric solid organ transplant (SOT) recipients is not available, several case reports show that pediatric SOT patients infected with COVID-19 showed only mild disease, even while on immunosuppressive therapy [25,26]. As children have different etiologies of ESRD, different comorbidities and seem to be affected differently by COVID-19, uniform policies affecting access to transplantation for both children and adults are not appropriate nor in the best interest of pediatric patients [16]. The increased mortality while on the waitlist that we showed in pediatric patients is a striking metric that supports the need for an individualized approach for pediatric KT patients. As pediatric KT events are relatively rare compared to adult KT, this affords an opportunity for patient- level versus center-level decisions about risk and benefit of KT in a pandemic setting.

The reduction of transplant events in children seen during the Middle (March 15 - April 30, 2020) COVID- 19 era was likely secondary to a combination of factors. As hospitals shifted resources to treat COVID-19 patients there was a decrease in available ICU beds for post-operative management and restrictions on operating room availability. Pediatric KT patients require intensive post-operative care, which may strain healthcare systems already overburdened by COVID-19 patients [27]. In addition, there was a notable decline in deceased donor organs recovered during March and April [28]. Shortages of COVID-19 testing or delayed results for deceased donors may have impacted center willingness to accept an organ from what would otherwise be an acceptable donor. As testing capacity increased across the country, this limitation was ameliorated. Finally, many centers stopped or significantly reduced elective and non-emergent surgical procedures, which likely had a significant impact on LDKT. For pediatric patients scheduled to receive an LDKT, it should be argued that transplant is not truly an elective procedure whether pre-emptive or not. Dialysis initiation would require at least one surgical procedure to establish dialysis access, and initiation or continuation of hemodialysis would result in a much higher COVID-19 exposure risk [29].

While peritoneal dialysis patients do not require frequent in-center visits and thereby can have minimal healthcare exposure, especially with the advancement of telehealth capabilities across the country, the benefit of transplant over dialysis has been well established in the pediatric ESRD population [30,31]. As healthcare centers lifted restrictions on elective cases and practice patterns changed, there was a large increase in LDKT in June 2020 suggesting cases had been postponed due to the pandemic.

Our hypothesis that children who received a transplant during the peak of the pandemic would differ in some characteristics compared to pre-pandemic patients was not supported. There was not a statistically significant difference in donor KDPI, recipient cPRA, or etiology of ESRD. While cold ischemia time in the Middle time period was longer, this is unlikely to be a clinically significant difference. This is the first study to describe characteristics of donors and recipients receiving a KT during the pandemic.

As this is a registry study, we are limited in the information that is available for analysis and are not able to delve into granular details of waitlist removal or patient death. While transplant centers have mandatory reporting requirements to UNOS/OPTN, data transmission may be delayed due to center practices and pandemic effect on workflow. Nevertheless, we are able to make generalized conclusions about the effects of COVID-19 on access to KT in pediatric patients. We were unable to differentiate regional variability in transplant rates and waitlist changes due to the overall low number of events in pediatric patients.

In summary, we found that the COVID-19 pandemic has had a significant impact on pediatric KT waitlist mortality, waitlist registration, DDKT and LDKT. Further studies to assess outcomes of pediatric patients who received a KT during this time are necessary to inform changes in policies and practices to optimize pediatric transplant outcomes and ensure access to this life-saving treatment.

## Figures and Tables

**Table 1.** Patient and donor characteristics broken down by three time periods of COVID-19 activity in 2020.

	Early Jan 1 – Mar 15	Middle Mar 15 – Apr 30	Late May 1 – Jun 30	p-value
N	122	36	121	
**Recipient Factors**				
Wait time (IQR)	202.5 (81, 618)	201.5 (42.5, 547)	227 (92, 495)	0.87
Female Sex	59 (48.4%)	14 (38.9%)	56 (46.3%)	0.61
Race				0.28
White	95 (77.9%)	21 (58.3%)	86 (71.1%)	
Black	16 (13.1%)	11 (30.6%)	25 (20.7%)	
Asian	7 (5.7%)	3 (8.3%)	8 (6.6%)	
Others	4 (3.3%)	1 (2.8%)	2 (1.7%)	
ABO Blood Type				0.94
Type O	70 (57.4%)	21 (58.3%)	71 (58.7%)	
Type A	30 (24.6%)	10 (27.8%)	34 (28.1%)	
Type B	16 (13.1%)	4 (11.1%)	13 (10.7%)	
Type AB	6 (4.9%)	1 (2.8%)	3 (2.5%)	
Primary diagnosis				0.065
Glomerulonephritis	21 (17.2%)	2 (5.6%)	17 (14.0%)	
FSGS	15 (12.3%)	5 (13.9%)	15 (12.4%)	
Hypoplasia	32 (26.2%)	7 (19.4%)	14 (11.6%)	
Obstructive	19 (15.6%)	8 (22.2%)	19 (15.7%)	
Polycystic	9 (7.4%)	2 (5.6%)	18 (14.9%)	
Others	26 (21.3%)	12 (33.3%)	38 (31.4%)	
cPRA, mean (SD)	12% (24)	9% (26)	7% (20)	0.29
cPRA ≠ 0, median (IQR)	44% (5, 64) (n=36)	61% (5, 99) (n=7)	11% (0, 52) (n=31)	0.087
cPRA ≥ 0.2	23 (18.9%)	4 (11.1%)	15 (12.4%)	0.33
**Donor Factors**				
Age (Yr), median (IQR)	28 (20, 34)	25.50 (21.50, 32)	28 (22, 35)	0.68
Female Gender	52 (42.6%)	16 (44.4%)	51 (42.1%)	0.97
Living donor	36 (29.5%)	**5 (13.9%)**	44 (36.4%)	**0.035**
Race				0.55
White	103 (84.4%)	29 (80.6%)	102 (84.3%)	
Black	13 (10.7%)	3 (8.3%)	15 (12.4%)	
Asian	3 (2.5%)	3 (8.3%)	3 (2.5%)	
Other race	3 (2.5%)	1 (2.8%)	1 (0.8%)	
KDPI	n=86	n=31	n=77	
median (IQR)	13.73 (7.65, 26.72)	14.57 (5.93, 28.99)	17.56 (9.71, 28.34)	0.46
mean (SD)	17.30 (13.46)	17.38 (12.63)	19.21 (12.18)	0.61
Cold Ischemic Time (Hours), median (IQR)	9.0 (4.0, 13.2)	10.2 (6.5, 17.5)	7.6 (2.4, 10.7)	**0.021**
	(n=121)	(n=34)	(n=57)	

**Table 2.** Observed compared to expected events in early and later COVID-19 eras of (A) waitlist changes, (B) transplant events

Waitlist changes (pediatric age at listing)		March 15– April 30, 2020				May 1 – June 30, 2020				Total			
		Observed	expected	% change	p value	observed	expected	% change	p value	observed	expected	% change	p value
New listing		132	131.5	0.4	0.967	130	170.7	−23.8	0.002	262	302.2	−13.3	0.021
Causes of removal	Death	6	2.1	189.0	0.006	3	2.7	11.3	0.852	9	4.8	88.7	0.053
	DDKT	42	64.5	−34.9	0.005	93	83.7	11.1	0.309	135	148.2	−8.9	0.279
	LDKT	5	27.8	−82.0	< 0.01	44	36.1	21.9	0.188	49	63.9	−23.3	0.062
Changed to inactive status		83	32.9	152.0	< 0.01	38	42.7	−11.1	0.468	119	75.7	57.2	< 0.01

Transplant (pediatric age at transplant)	March 15– April 30, 2020				May 1 – June 30, 2020				Total			
	Observed	expected	% change	p value	observed	expected	% change	p value	observed	expected	% change	p value
Total transplant	36	88.5	−59.3	< 0.01	121	114.8	5.4	0.566	157	203.3	−22.8	0.001
LDKT	5	27.9	−82.1	< 0.01	44	36.3	21.3	0.199	49	64.2	−23.7	0.058
DDKT	31	59.1	−47.6	< 0.01	77	76.8	0.3	1.000	108	135.9	−20.5	0.017
DCD	1	3.2	−68.4	0.224	5	4.1	21.6	0.663	6	7.3	−17.6	0.632
Regional or national import	7	3.7	90.7	0.082	4	4.8	−16.1	0.729	11	8.4	30.4	0.377

**Table 3.** Observed center-level events as a proportion of expected events, March 15 -June 30, 2020. **Bold** denotes IRRs that are statistically significantly different from the IRR in states with low COVID-19 disease burden (<8000 cases PMP).

COVID-19 rates	New listings	DDKT	LDKT	Waitlist death
Overall	0.77 0.87 0.98	0.66 0.79 0.96	0.58 0.76 1.01	0.98 1.89 3.63
Low	0.82 0.94 1.08	0.66 0.81 1.00	0.66 0.89 1.20	0.84 1.87 4.16
High*	**0.49 0.65 0.85**	0.48 0.73 1.12	**0.17 0.38 0.84**	0.62 1.92 5.96

* States with high COVID-19 burden: NY, NJ, RI, MA, DC, CT, LA, DE, IL, MD, AZ, NE, IA, NS

## Abbreviations

COVID-19: coronavirus disease 2020
KT: kidney transplant
LDKT: ive donor kidney transplantation
DDKT: deceased donor transplantation
ESRD: end stage renal disease
SRTR: Scientific Registry of Transplant Recipients
OPTN: Organ Procurement and Transplantation Network
PMP: per million population
DCD: donation after circulatory death
IRR: incidence rate ratio
ICU: intensive care unit
OR: operating room
